# 1-(Hex-5-en-1-yl)-4-{[3-methyl-2,3-di­hydro-1,3-benzo­thia­zol-2-yl­idene]meth­yl}quinolin-1-ium iodide monohydrate

**DOI:** 10.1107/S2414314622007970

**Published:** 2022-08-16

**Authors:** Nathaniel Shank, Andrea L. Stadler, Sean P. Barrett, Clifford W. Padgett

**Affiliations:** a Department of Chemistry and Biochemistry, Georgia Southern University, Armstrong Campus, 11935 Abercorn Street, Savannah GA 31419, USA; b Chemistry, Department of Physical Sciences, St. Joseph’s College, 155 West Roe Blvd, Patchogue, NY 11772, USA; University of Aberdeen, Scotland

**Keywords:** crystal structure, π–stacking, thia­zole orange

## Abstract

The title thia­zole orange derivative, bearing an alkene substituent, crystallized as a monohydrate of its iodide salt, namely, (*Z*)-1-(hex-5-en-1-yl)-4-{[3-methyl-2,3-di­hydro-1,3-benzo­thia­zol-2-yl­idene]meth­yl}quinolin-1-ium iodide monohydrate, C_24_H_25_N_2_S^+^·I^−^·H_2_O. The packing features aromatic π-stacking and van der Waals inter­actions. The water mol­ecule of crystallization inter­acts with the cation and anion *via* O—H⋯N and O—H⋯I hydrogen bonds, respectively.

## Abstract 2

The structure of 4-hexenyl thia­zole orange is presented.

## Graphical abstract

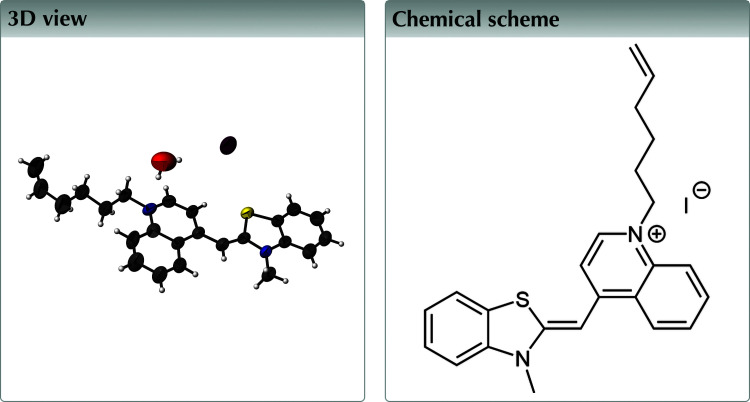

## Structure description

Inter­calating dyes are a standard means to detect duplex DNA or RNA *in vitro* and *in vivo*. The cyanine dye thia­zole orange has been used extensively as a on/off fluorescent probe in a host of biological applications (Suss *et al.*, 2021 ▸). The *bis*-inter­calating dye based on thia­zole orange has been shown to have an increased affinity towards duplexed oligomers and retains its fluoro­genic characteristic (Rye *et al.*, 1992 ▸). In an effort to enhance the binding affinity further, and essentially create a non-covalent inter­action that is effectively permanent, we synthesized a thia­zole orange dye bearing an alkene substituent that is capable of participating in polymerization reactions. Access to polymeric thia­zole orange dye and other cyanine dyes will afford extremely bright, highly organized, and versatile fluorescent probes that can be attached to mol­ecules of inter­est and mitigate the equilibrium the dye would establish with endogenous duplexes.

**Figure 1:**
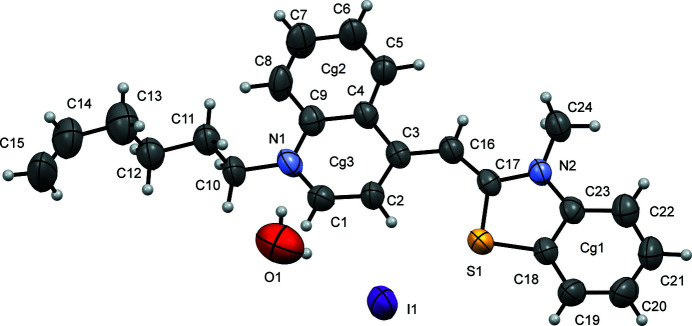
A view of the title compound, showing the atom labeling. Displacement ellipsoids are drawn at the 50% probability level.

Herein we report the crystal structure of 4-hexenyl thia­zole orange iodide monohydrate, C_24_H_25_N_2_S^+^·I^−^·H_2_O, which crystallizes in the triclinic space group *P* 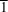 . In the cation (Fig. 1 ▸), the benzo­thia­zole ring is titled by 3.32 (13)° with respect to the quinoline ring system: as a result the mol­ecule is close to planar (excluding the hex-1-ene group) with an r.m.s. deviation of 0.048 Å for the non-hydrogen atoms; including the hex-1-ene group increases the r.m.s.d to 0.416 Å for the non-hydrogen atoms. The crystal structure contains a water mol­ecule of crystallization bound to the cation *via* a weak O1—H1*A*⋯N1 hydrogen bond [O⋯N = 3.014 (10) Å] and the anion *via* an O1—H1*B*⋯I1 link [O1⋯I1 = 3.546 (10) Å] (Table 1 ▸). There is also a weak C2—H2⋯S1 intra­molecular inter­action with C⋯S = 3.128 (7) that helps to maintain the coplanarity of the two ring systems.

**Figure 2:**
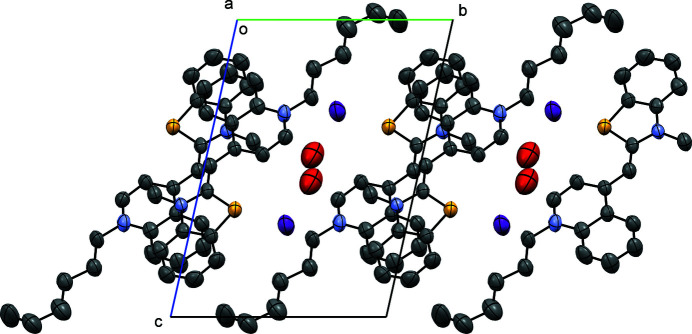
Crystal packing diagram of the title compound viewed down the *b*-axis direction with H atoms omitted for clarity.

In the extended structure (Fig. 2 ▸), aromatic π–π stacking is observed with *Cg*1⋯*Cg*2^i^ = 3.559 (6) Å [symmetry code: (i) 2 − *x*, 2 − *y*, 1 − *z*] and *Cg*1⋯*Cg*3^i^ = 3.492 (5) Å, where *Cg*1 is the centroid of the phenyl ring of the benzo­thia­zole group containing atoms C18–C23, *Cg*2 is the centroid of the phenyl ring of the quinoline group containing atoms C4–C9, and *Cg*3 is the centroid of the pyridyl ring of the quinoline groups containing atoms N1/C1–C4/C9. These π–stacking inter­actions run along the [100] direction with neighboring layers held together with van der Waals inter­actions.

## Synthesis and crystallization

**Figure 3:**
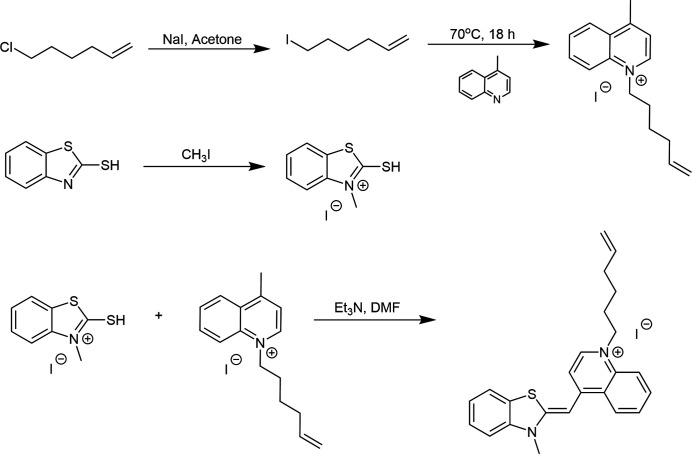
Reaction scheme.

All materials were purchased from Fisher Scientific or Sigma Aldrich and used as received. All flash chromatography was performed with 230 × 400 mesh silica gel. Pure samples were analyzed with a Joel 300 MHz NMR and HRMS of the title compound was acquired on a Shimadzu LCMS 9030 QTof operating in positive mode. The reaction scheme is shown in Fig. 3 ▸.

**6-Iodo­hex-1-ene (1)**

In a conical reaction vial with a magnetic stir bar, 3.0 g of 6-chloro­hex-1-ene (25.4 mmol, 1 eqv) was dissolved in 50 ml of acetone. To this solution was added 11.36 g (76.3 mmol, 3 equiv.) of sodium iodide. The solution was warmed slightly to assist with dissolving the sodium iodide and then covered and stirred for 48 h. An equal portion of hexane was added to the reaction and then the solids were filtered. The volatiles were stripped and the product was purified on silica with 100% hexa­nes as the eluent. Yield 3.31 g (62%) NMR: ^1^H NMR [300 MHz, (CDCl_3_] δ = 5.77 (*m*, 1H, –C**H**=CH_2_), 4.98 (*m*, 2H, –CH=C**H_2_**), 2.19 (*t*, 2H, –C**H_2_** I), 2.07 (*t*, 2H, –CH_2_C**H_2_** CH_2_I), 1.77 (*t*, 2H, –C**H_2_** CH_2_CH_2_I), 1.52 (*t*, 2H, –CH_2_= CHC**H_2_** CH_2_) p.p.m.

**1-(Hex-5-en-1-yl)-4-methyl­quinolin-1-ium iodide (2)**

To a conical reaction vial with a magnetic stir bar was added 0.22 g (1.58 mmol, 1 eqv) of 4-methyl­quinoline and 0.5 g (2.38 mmol, 1.5 equiv.) of 6-iodo­hex-1-ene. The reaction was stirred at 70°C for 18 h. The reaction was then purified on silica eluting with 2% methanol in DCM. Yield 0.54 g (96%) NMR: ^1^H NMR [300 MHz, (CDCl_3_)] δ = 10.17 (*d*, 1H, Ar.), 8.37 (*m*, 2H, Ar.), 8.20 (*t*, 1H, Ar.), 8.01 (*m*, 2H, Ar.), 5.71 (*m*, 1H, C**H**=CH_2_), 5.28 (*t*, 2H, –C**H_2_** N), 4.96 (*m*, 2H, –CH=C**H_2_**), 2.12 (*m*, 4H, –C**H_2_** C**H**_2_CH_2_), 1.62 (*t*, 2H, –CH_2_= CHC**H_2_** CH_2_) p.p.m.

**2-Mercapto-3-methyl­benzo­thia­zol-3-ium iodide (3)**

To a conical reaction flask was added 1 g (6.0 mmol, 1 eqv) of benzo­thia­zole-2-thiol and 2.2 g (15.5 mmol, 2.6 eqv) of methyl iodide. The reaction was allowed to stir at 50°C for 24 h and then taken up in a minimal amount of methanol. The concentrated solution was then titrated into ether to form a precipitate that was collected by filtration. This provided the product as a white solid that needed no further purification. Yield 0.75 g (69%) NMR: ^1^H NMR [300 MHz, (CD_3_)_2_SO] δ = 8.43 (*d*, 1H, Ar.), 8.29 (*d*, 1H, Ar.), 7.90 (*t*, 2H, Ar.), 7.80 (*t*, 2H, Ar.), 4.20 (*s*, 3H, –C**H_3_**), 3.17 (*t*, 3H, –SC**H_3_**) p.p.m.

**(** ***Z*** **)-1-(Hex-5-en-1-yl)-4-((3-methyl­benzo[** ***d*** **]thia­zol-2(3** ***H*** **)-yl­idene)meth­yl)quinolin-1-ium iodide (4)**

Into a conical reaction vial with a magnetic stir bar was added 106 mg (0.3 mmol, 1 eqv) of **2** that was dissolved in 2 ml of DMF. A total of 97 mg (0.3 mmol, 1 eqv) of **3** was added followed by the addition of 42 mg (0.3 mmol, 1 equiv.) of tri­ethyl­amine. The solution immediately turned dark red and was allowed to stir for 48 h.

The solution was then added to ether, and the orange solid was collected.

The title compound was then purified using a gradient (2–5%) of methanol in DCM. Yield 45 mg (30%). NMR: ^1^H NMR [300 MHz, (CD_3_)_2_SO)] δ = 8.80 (*d*, 1H, Ar.), 8.63 (*d*, 1H, Ar.), 8.15 (*d*, 1H, Ar.), 8.06 (*d*, 1H, Ar.), 7.99 (*t*, 1H, Ar.), 7.77 (*q*, 2H, Ar.), 7.62 (*t*, 1H, Ar.), 7.40 (*m*, 2H, Ar.), 5.77 (*m*, 1H, –C**H**=CH_2_), 4.97 (*t*, 2H, –C**H_2_** N), 4.61 (*t*, 2H, –CH=**CH_2_**), 4.02 (*s*, 3H, –N—C**H_3_**) 2.08 (*q*, 2H, –CH_2_=CHC**H_2_** CH_2_), 1.85 (*quin*, 2H, –CH_2_C**H_2_** CH_2_), 1.45 (*t*, 2H, –CH_2_CH_2_CH_2_–) p.p.m.

Crystal formation: the title compound was taken up in methanol and then allowed to crystallize as dark-red prisms by slow evaporation of the solvent.

## Refinement

Crystal data, data collection and structure refinement details are summarized in Table 2 ▸.

## Supplementary Material

Crystal structure: contains datablock(s) I, global. DOI: 10.1107/S2414314622007970/hb4408sup1.cif

Structure factors: contains datablock(s) I. DOI: 10.1107/S2414314622007970/hb4408Isup2.hkl

CCDC reference: 2195631

Additional supporting information:  crystallographic information; 3D view; checkCIF report

## Figures and Tables

**Table 1:** Hydrogen-bond geometry (Å, °)

*D*—H⋯*A*	*D*—H	H⋯*A*	*D*⋯*A*	*D*—H⋯*A*
C2—H2⋯S1	0.93	2.40	3.128 (7)	135
O1—H1*A*⋯N1	0.85	2.39	3.014 (10)	131
O1—H1*B*⋯I1	0.85	2.71	3.546 (10)	169

**Table 2:** Experimental details

Crystal data	
Chemical formula	C_24_H_25_N_2_S^+^·I^−^·H_2_O
*M*_r_	518.43
Crystal system, space group	Triclinic, *P* 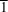
Temperature (K)	170
*a*, *b*, *c* (Å)	8.4780 (11), 10.5773 (17), 14.5191 (19)
α, β, γ (°)	95.810 (12), 105.762 (12), 110.651 (14)
*V* (Å^3^)	1144.1 (3)
*Z*	2
Radiation type	Mo *K*α
μ (mm^−1^)	1.51
Crystal size (mm)	0.5 × 0.1 × 0.1

Data collection	
Diffractometer	XtaLAB Mini (ROW)
Absorption correction	Multi-scan (*CrysAlis PRO*; Rigaku OD, 2019 ▸)
*T*_min_, *T*_max_	0.332, 1.000
No. of measured, independent and observed [*I* > 2σ(*I*)] reflections	6581, 4189, 2249
*R*_int_	0.043
(sin θ/λ)_max_ (Å^−1^)	0.602

Refinement	
*R*[*F* ^2^ > 2σ(*F* ^2^)], *wR*(*F* ^2^), *S*	0.060, 0.171, 1.03
No. of reflections	4189
No. of parameters	266
H-atom treatment	H-atom parameters constrained
Δρ_max_, Δρ_min_ (e Å^−3^)	0.66, −0.60

Computer programs: *CrysAlis PRO* (Rigaku OD, 2019 ▸), *SHELXT* (Sheldrick, 2015*a* ▸), *SHELXL2018/1* (Sheldrick, 2015*b* ▸), and *OLEX2* (Dolomanov *et al.*, 2009 ▸).

## full crystallographic data

### Crystal data

**Table:**

C_24_H_25_N_2_S^+^·I^−^·H_2_O	*Z* = 2
*M**_r_* = 518.43	*F*(000) = 524
Triclinic, *P*1	*D*_x_ = 1.505 Mg m^−^^3^
*a* = 8.4780 (11) Å	Mo *K*α radiation, λ = 0.71073 Å
*b* = 10.5773 (17) Å	Cell parameters from 644 reflections
*c* = 14.5191 (19) Å	θ = 2.1–21.1°
α = 95.810 (12)°	µ = 1.51 mm^−^^1^
β = 105.762 (12)°	*T* = 170 K
γ = 110.651 (14)°	Rect. prism, clear dark red
*V* = 1144.1 (3) Å^3^	0.5 × 0.1 × 0.1 mm

### Data collection

**Table:**

XtaLAB Mini (ROW) diffractometer	4189 independent reflections
Radiation source: fine-focus sealed X-ray tube, Rigaku (Mo) X-ray Source	2249 reflections with *I* > 2σ(*I*)
Graphite monochromator	*R*_int_ = 0.043
ω scans	θ_max_ = 25.4°, θ_min_ = 2.1°
Absorption correction: multi-scan (CrysAlisPro; Rigaku OD, 2019)	*h* = −10→10
*T*_min_ = 0.332, *T*_max_ = 1.000	*k* = −12→11
6581 measured reflections	*l* = −17→16

### Refinement

**Table:**

Refinement on *F*^2^	Primary atom site location: dual
Least-squares matrix: full	Hydrogen site location: mixed
*R*[*F*^2^ > 2σ(*F*^2^)] = 0.060	H-atom parameters constrained
*wR*(*F*^2^) = 0.171	*w* = 1/[σ^2^(*F*_o_^2^) + (0.0622*P*)^2^ + 0.1317*P*] where *P* = (*F*_o_^2^ + 2*F*_c_^2^)/3
*S* = 1.03	(Δ/σ)_max_ < 0.001
4189 reflections	Δρ_max_ = 0.66 e Å^−^^3^
266 parameters	Δρ_min_ = −0.60 e Å^−^^3^
0 restraints	

### Special details

**Table:**

Geometry. All esds (except the esd in the dihedral angle between two l.s. planes) are estimated using the full covariance matrix. The cell esds are taken into account individually in the estimation of esds in distances, angles and torsion angles; correlations between esds in cell parameters are only used when they are defined by crystal symmetry. An approximate (isotropic) treatment of cell esds is used for estimating esds involving l.s. planes.
Refinement. All H atoms were placed in idealized locations (C—H = 0.93–0.97, O—H = 0.85 Å) and refined as riding atoms.

### Fractional atomic coordinates and isotropic or equivalent isotropic displacement parameters (Å^2^)

**Table:**

	*x*	*y*	*z*	*U*_iso_*/*U*_eq_	
I1	0.04034 (9)	0.55965 (6)	0.30846 (5)	0.0920 (3)	
S1	0.7927 (3)	0.81364 (17)	0.36094 (14)	0.0577 (5)	
N2	0.8314 (7)	1.0670 (5)	0.3757 (4)	0.0495 (14)	
N1	0.6019 (8)	0.6852 (5)	0.6841 (5)	0.0579 (15)	
C23	0.8637 (9)	1.0327 (7)	0.2901 (5)	0.0511 (17)	
C4	0.6594 (9)	0.9170 (7)	0.6522 (5)	0.0498 (17)	
C16	0.7461 (9)	0.9748 (7)	0.5082 (5)	0.0537 (17)	
H16	0.746994	1.060734	0.530348	0.064*	
C3	0.7026 (9)	0.8785 (6)	0.5662 (5)	0.0486 (17)	
C17	0.7876 (8)	0.9617 (6)	0.4235 (5)	0.0467 (16)	
C9	0.6078 (9)	0.8171 (7)	0.7091 (5)	0.0549 (18)	
C2	0.6923 (9)	0.7436 (7)	0.5470 (5)	0.0559 (18)	
H2	0.716781	0.713601	0.492143	0.067*	
C1	0.6479 (9)	0.6548 (7)	0.6053 (5)	0.0590 (19)	
H1	0.649126	0.567645	0.590268	0.071*	
C18	0.8509 (9)	0.8971 (7)	0.2716 (5)	0.0544 (18)	
C22	0.9072 (10)	1.1178 (8)	0.2252 (6)	0.066 (2)	
H22	0.918042	1.209023	0.236988	0.080*	
C10	0.5465 (10)	0.5769 (7)	0.7392 (6)	0.067 (2)	
H10A	0.515625	0.487592	0.698316	0.080*	
H10B	0.439612	0.576184	0.751890	0.080*	
C5	0.6653 (10)	1.0472 (7)	0.6860 (5)	0.0609 (19)	
H5	0.701850	1.115918	0.652057	0.073*	
C11	0.6831 (11)	0.5937 (7)	0.8340 (6)	0.066 (2)	
H11A	0.705991	0.678282	0.878000	0.079*	
H11B	0.793698	0.601946	0.822871	0.079*	
C6	0.6208 (11)	1.0797 (8)	0.7658 (6)	0.072 (2)	
H6	0.624418	1.167804	0.783995	0.086*	
C24	0.8400 (10)	1.2008 (7)	0.4111 (6)	0.064 (2)	
H24A	0.927482	1.239531	0.475373	0.096*	
H24B	0.725376	1.193066	0.414159	0.096*	
H24C	0.872977	1.259910	0.367520	0.096*	
C8	0.5616 (11)	0.8527 (8)	0.7912 (6)	0.071 (2)	
H8	0.524265	0.786504	0.826843	0.085*	
O1	0.2316 (12)	0.5102 (8)	0.5443 (7)	0.138 (3)	
H1A	0.291275	0.581291	0.590880	0.207*	
H1B	0.192515	0.534755	0.491739	0.207*	
C19	0.8832 (11)	0.8450 (8)	0.1895 (6)	0.073 (2)	
H19	0.877847	0.755232	0.177902	0.087*	
C12	0.6232 (11)	0.4721 (8)	0.8818 (6)	0.071 (2)	
H12A	0.512182	0.464089	0.892323	0.085*	
H12B	0.599653	0.387829	0.837227	0.085*	
C20	0.9230 (12)	0.9296 (9)	0.1268 (6)	0.081 (2)	
H20	0.943321	0.895594	0.071436	0.097*	
C21	0.9340 (11)	1.0618 (9)	0.1423 (6)	0.075 (2)	
H21	0.959534	1.115594	0.097174	0.090*	
C7	0.5706 (12)	0.9825 (9)	0.8191 (7)	0.080 (3)	
H7	0.542670	1.005128	0.874551	0.095*	
C13	0.7538 (14)	0.4843 (11)	0.9753 (8)	0.110 (3)	
H13A	0.770531	0.565495	1.020756	0.132*	
H13B	0.867061	0.500098	0.965176	0.132*	
C14	0.7080 (18)	0.3633 (12)	1.0222 (8)	0.113 (4)	
H14	0.790099	0.376037	1.083381	0.136*	
C15	0.585 (2)	0.2529 (12)	0.9957 (10)	0.146 (6)	
H15A	0.496588	0.231168	0.935308	0.175*	
H15B	0.578332	0.188806	1.035348	0.175*	

### Atomic displacement parameters (Å^2^)

**Table:**

	*U*^11^	*U*^22^	*U*^33^	*U*^12^	*U*^13^	*U*^23^
I1	0.1378 (6)	0.0665 (4)	0.0968 (5)	0.0488 (4)	0.0612 (4)	0.0317 (3)
S1	0.0704 (12)	0.0407 (9)	0.0605 (12)	0.0162 (9)	0.0271 (10)	0.0111 (9)
N2	0.057 (4)	0.041 (3)	0.055 (4)	0.019 (3)	0.024 (3)	0.018 (3)
N1	0.058 (4)	0.041 (3)	0.072 (4)	0.011 (3)	0.029 (3)	0.016 (3)
C23	0.045 (4)	0.048 (4)	0.056 (5)	0.010 (3)	0.019 (4)	0.014 (4)
C4	0.053 (4)	0.043 (4)	0.061 (5)	0.019 (3)	0.028 (4)	0.017 (3)
C16	0.060 (5)	0.046 (4)	0.061 (5)	0.021 (4)	0.028 (4)	0.017 (4)
C3	0.048 (4)	0.043 (4)	0.055 (4)	0.016 (3)	0.018 (3)	0.016 (3)
C17	0.038 (4)	0.042 (4)	0.058 (5)	0.010 (3)	0.018 (3)	0.014 (3)
C9	0.050 (4)	0.058 (4)	0.064 (5)	0.024 (4)	0.025 (4)	0.020 (4)
C2	0.069 (5)	0.048 (4)	0.057 (5)	0.019 (4)	0.033 (4)	0.018 (4)
C1	0.069 (5)	0.047 (4)	0.060 (5)	0.018 (4)	0.027 (4)	0.008 (4)
C18	0.057 (5)	0.047 (4)	0.055 (5)	0.013 (4)	0.023 (4)	0.013 (4)
C22	0.066 (5)	0.068 (5)	0.075 (6)	0.028 (4)	0.033 (4)	0.027 (5)
C10	0.084 (6)	0.050 (4)	0.070 (5)	0.016 (4)	0.042 (5)	0.024 (4)
C5	0.078 (5)	0.057 (4)	0.062 (5)	0.031 (4)	0.035 (4)	0.022 (4)
C11	0.080 (5)	0.059 (5)	0.073 (6)	0.030 (4)	0.039 (5)	0.024 (4)
C6	0.101 (6)	0.062 (5)	0.083 (6)	0.045 (5)	0.059 (5)	0.026 (4)
C24	0.073 (5)	0.054 (4)	0.077 (6)	0.027 (4)	0.036 (4)	0.027 (4)
C8	0.088 (6)	0.078 (5)	0.082 (6)	0.043 (5)	0.060 (5)	0.042 (5)
O1	0.121 (7)	0.111 (6)	0.159 (8)	0.044 (6)	0.020 (6)	0.009 (5)
C19	0.091 (6)	0.058 (5)	0.066 (5)	0.015 (5)	0.043 (5)	0.006 (4)
C12	0.081 (6)	0.063 (5)	0.078 (6)	0.028 (5)	0.039 (5)	0.025 (5)
C20	0.093 (7)	0.078 (6)	0.068 (6)	0.016 (5)	0.048 (5)	0.009 (5)
C21	0.084 (6)	0.072 (5)	0.070 (6)	0.017 (5)	0.040 (5)	0.028 (5)
C7	0.106 (7)	0.080 (6)	0.096 (7)	0.057 (6)	0.069 (6)	0.035 (5)
C13	0.123 (9)	0.109 (8)	0.108 (8)	0.051 (7)	0.039 (7)	0.052 (7)
C14	0.159 (11)	0.105 (8)	0.094 (8)	0.060 (9)	0.051 (8)	0.051 (7)
C15	0.259 (18)	0.093 (8)	0.128 (11)	0.065 (11)	0.129 (12)	0.048 (8)

### Geometric parameters (Å, º)

**Table:**

S1—C17	1.750 (7)	C11—H11A	0.9700
S1—C18	1.726 (7)	C11—H11B	0.9700
N2—C23	1.383 (8)	C11—C12	1.518 (9)
N2—C17	1.366 (8)	C6—H6	0.9300
N2—C24	1.426 (8)	C6—C7	1.367 (10)
N1—C9	1.385 (8)	C24—H24A	0.9600
N1—C1	1.349 (9)	C24—H24B	0.9600
N1—C10	1.478 (8)	C24—H24C	0.9600
C23—C18	1.392 (9)	C8—H8	0.9300
C23—C22	1.400 (10)	C8—C7	1.361 (10)
C4—C3	1.453 (9)	O1—H1A	0.8500
C4—C9	1.427 (9)	O1—H1B	0.8499
C4—C5	1.393 (9)	C19—H19	0.9300
C16—H16	0.9300	C19—C20	1.365 (11)
C16—C3	1.399 (9)	C12—H12A	0.9700
C16—C17	1.375 (9)	C12—H12B	0.9700
C3—C2	1.392 (9)	C12—C13	1.461 (11)
C9—C8	1.408 (10)	C20—H20	0.9300
C2—H2	0.9300	C20—C21	1.360 (11)
C2—C1	1.351 (9)	C21—H21	0.9300
C1—H1	0.9300	C7—H7	0.9300
C18—C19	1.397 (10)	C13—H13A	0.9700
C22—H22	0.9300	C13—H13B	0.9700
C22—C21	1.396 (11)	C13—C14	1.491 (13)
C10—H10A	0.9700	C14—H14	0.9300
C10—H10B	0.9700	C14—C15	1.196 (15)
C10—C11	1.485 (10)	C15—H15A	0.9300
C5—H5	0.9300	C15—H15B	0.9300
C5—C6	1.360 (10)		
			
C18—S1—C17	91.7 (3)	C10—C11—C12	111.7 (7)
C23—N2—C24	123.1 (5)	H11A—C11—H11B	107.9
C17—N2—C23	114.8 (5)	C12—C11—H11A	109.3
C17—N2—C24	122.1 (6)	C12—C11—H11B	109.3
C9—N1—C10	123.0 (6)	C5—C6—H6	120.1
C1—N1—C9	118.1 (6)	C5—C6—C7	119.9 (7)
C1—N1—C10	118.9 (6)	C7—C6—H6	120.1
N2—C23—C18	112.7 (6)	N2—C24—H24A	109.5
N2—C23—C22	127.5 (6)	N2—C24—H24B	109.5
C18—C23—C22	119.8 (7)	N2—C24—H24C	109.5
C9—C4—C3	119.4 (6)	H24A—C24—H24B	109.5
C5—C4—C3	125.1 (6)	H24A—C24—H24C	109.5
C5—C4—C9	115.5 (7)	H24B—C24—H24C	109.5
C3—C16—H16	115.1	C9—C8—H8	119.5
C17—C16—H16	115.1	C7—C8—C9	121.1 (7)
C17—C16—C3	129.9 (6)	C7—C8—H8	119.5
C16—C3—C4	119.3 (6)	H1A—O1—H1B	109.5
C2—C3—C4	115.6 (6)	C18—C19—H19	121.0
C2—C3—C16	125.1 (7)	C20—C19—C18	118.1 (7)
N2—C17—S1	110.0 (5)	C20—C19—H19	121.0
N2—C17—C16	123.3 (6)	C11—C12—H12A	108.9
C16—C17—S1	126.8 (5)	C11—C12—H12B	108.9
N1—C9—C4	120.7 (7)	H12A—C12—H12B	107.7
N1—C9—C8	119.7 (6)	C13—C12—C11	113.6 (7)
C8—C9—C4	119.6 (6)	C13—C12—H12A	108.9
C3—C2—H2	118.8	C13—C12—H12B	108.9
C1—C2—C3	122.4 (7)	C19—C20—H20	118.8
C1—C2—H2	118.8	C21—C20—C19	122.4 (8)
N1—C1—C2	123.8 (7)	C21—C20—H20	118.8
N1—C1—H1	118.1	C22—C21—H21	119.6
C2—C1—H1	118.1	C20—C21—C22	120.7 (8)
C23—C18—S1	110.8 (5)	C20—C21—H21	119.6
C23—C18—C19	120.8 (7)	C6—C7—H7	120.0
C19—C18—S1	128.3 (5)	C8—C7—C6	120.0 (8)
C23—C22—H22	120.9	C8—C7—H7	120.0
C21—C22—C23	118.2 (7)	C12—C13—H13A	108.3
C21—C22—H22	120.9	C12—C13—H13B	108.3
N1—C10—H10A	108.6	C12—C13—C14	116.0 (9)
N1—C10—H10B	108.6	H13A—C13—H13B	107.4
N1—C10—C11	114.8 (6)	C14—C13—H13A	108.3
H10A—C10—H10B	107.5	C14—C13—H13B	108.3
C11—C10—H10A	108.6	C13—C14—H14	114.1
C11—C10—H10B	108.6	C15—C14—C13	131.8 (13)
C4—C5—H5	118.1	C15—C14—H14	114.1
C6—C5—C4	123.9 (7)	C14—C15—H15A	120.0
C6—C5—H5	118.1	C14—C15—H15B	120.0
C10—C11—H11A	109.3	H15A—C15—H15B	120.0
C10—C11—H11B	109.3		
			
S1—C18—C19—C20	178.2 (6)	C9—C4—C3—C2	−1.0 (10)
N2—C23—C18—S1	1.5 (8)	C9—C4—C5—C6	−1.9 (12)
N2—C23—C18—C19	−178.5 (6)	C9—C8—C7—C6	1.7 (14)
N2—C23—C22—C21	−179.8 (7)	C1—N1—C9—C4	0.6 (10)
N1—C9—C8—C7	178.2 (7)	C1—N1—C9—C8	−179.7 (6)
N1—C10—C11—C12	−174.8 (6)	C1—N1—C10—C11	104.1 (8)
C23—N2—C17—S1	1.7 (7)	C18—S1—C17—N2	−0.7 (5)
C23—N2—C17—C16	−178.1 (6)	C18—S1—C17—C16	179.2 (6)
C23—C18—C19—C20	−1.7 (12)	C18—C23—C22—C21	1.0 (11)
C23—C22—C21—C20	−1.9 (12)	C18—C19—C20—C21	0.8 (14)
C4—C3—C2—C1	−1.1 (11)	C22—C23—C18—S1	−179.1 (5)
C4—C9—C8—C7	−2.1 (12)	C22—C23—C18—C19	0.8 (11)
C4—C5—C6—C7	1.6 (13)	C10—N1—C9—C4	−178.9 (6)
C16—C3—C2—C1	−179.3 (7)	C10—N1—C9—C8	0.8 (11)
C3—C4—C9—N1	1.2 (10)	C10—N1—C1—C2	176.7 (7)
C3—C4—C9—C8	−178.5 (7)	C10—C11—C12—C13	180.0 (8)
C3—C4—C5—C6	178.7 (7)	C5—C4—C3—C16	−3.2 (11)
C3—C16—C17—S1	1.7 (11)	C5—C4—C3—C2	178.4 (7)
C3—C16—C17—N2	−178.5 (7)	C5—C4—C9—N1	−178.2 (6)
C3—C2—C1—N1	3.1 (12)	C5—C4—C9—C8	2.1 (10)
C17—S1—C18—C23	−0.5 (6)	C5—C6—C7—C8	−1.4 (14)
C17—S1—C18—C19	179.6 (7)	C11—C12—C13—C14	−175.8 (9)
C17—N2—C23—C18	−2.1 (8)	C24—N2—C23—C18	178.8 (6)
C17—N2—C23—C22	178.6 (7)	C24—N2—C23—C22	−0.5 (11)
C17—C16—C3—C4	−178.6 (7)	C24—N2—C17—S1	−179.2 (5)
C17—C16—C3—C2	−0.3 (12)	C24—N2—C17—C16	1.0 (10)
C9—N1—C1—C2	−2.8 (11)	C19—C20—C21—C22	1.0 (14)
C9—N1—C10—C11	−76.4 (9)	C12—C13—C14—C15	3 (2)
C9—C4—C3—C16	177.4 (6)		

### Hydrogen-bond geometry (Å, º)

**Table:**

*D*—H···*A*	*D*—H	H···*A*	*D*···*A*	*D*—H···*A*
C2—H2···S1	0.93	2.40	3.128 (7)	135
O1—H1*A*···N1	0.85	2.39	3.014 (10)	131
O1—H1*B*···I1	0.85	2.71	3.546 (10)	169
